# Percutaneous Alcohol Sclerotherapy of Simple Hepatic Cysts. Results From a Multicentre Survey in Italy

**DOI:** 10.1155/1994/10372

**Published:** 1994

**Authors:** Marco Montorsi, Guido Torzilli, Uberto Fumagalli, Stefano Bona, Riccardo Rosati, Matilde de Simone, Vittorio Rovati, Franco Mosca, Carlo Filice

**Affiliations:** 1 Institute of General Surgery University of Milano-Ospedale Maggiore Policlinico I.R.C.C.S.-Milano Italy; 2 Surgical Pathology University of Milano-Ospedale L. Sacco-Milano Italy; 3 Institute of General Surgery University of Pisa-Ospedale Cisanello-Pisa Italy; 4 Institute of Infectious Diseases University of Pavia-Ospedale Policlinico San Matteo I.R.C.C.S.-Pavia Italy

## Abstract

The increased use of Ultrasonography (US) has led to increased detection of simple hepatic cysts. For symptomatic cysts treatment is necessary. Until some years ago surgery was the only therapy. We have treated a large number of patients with Percutaneous Alcohol Sclerotherapy (PAS) and evaluated retrospectively the efficacy of this approach.

Data on 21 patients with symptomatic simple hepatic cysts were reviewed retrospectively. Cysts had a mean diameter of 9 cm (range: 7–15 cm). PAS was always performed under local anesthesia and US guidance. 25% of the volume was replaced with 95% ethanol and then completely aspirated after 20–30 minutes.

No complications or deaths occurred. In all patients symptoms disappeared after treatment. In 15 out of 21 cases there was no evidence of residual cyst on US, computed tomography (CT) or magnetic resonance (MRI). In 6 patients with shorter follow-up, cysts showed a mean reduction in diameter of 50%. The mean follow-up was 18 months (range 6–60 months).

We conclude that PAS is easy with low risk for the patients and with good long-term results; it should therefore become the procedure of choice for simple hepatic cysts.


*HPB Surgery*, 1994, Vol. 8, pp. 89–94
Reprints available directly from the publisher
Photocopying permitted by license only

© 1994 Harwood Academic Publishers GmbH
Printed in Malaysia

### Percutaneous Alcohol Sclerotherapy of Simple Hepatic Cysts. Results From A Multicentre Survey in Italy

MARCO MONTORSI, GUIDO TORZILLI, UBERTO FUMAGALLI, STEFANO BONA, RICCARDO ROSATI, MATILDE DE SIMONE, VITTORIO ROVATI,* FRANCO MOSCA,# and CARLO FILICE†

Institute of General Surgery, University of Milano-Ospedale Maggiore Policlinico, I.R.C.C.S.-Milano, Italy
*Surgical Pathology, University of Milano-Ospedale L. Sacco-Milano
#Institute of General Surgery, University of Pisa-Ospedale Cisanello-Pisa
†Institute of Infectious Diseases, University of Pavia-Ospedale Policlinico San Matteo, I.R.C.C.S.-Pavia

The increased use of Ultrasonography (US) has led to increased detection of simple hepatic cysts. For symptomatic cysts treatment is necessary. Until some years ago surgery was the only therapy. We have treated a large number of patients with Percutaneous Alcohol Sclerotherapy (PAS) and evaluated retrospectively the efficacy of this approach.

Data on 21 patients with symptomatic simple hepatic cysts were reviewed retrospectively. Cysts had a mean diameter of 9 cm (range: 7–15 cm). PAS was always performed under local anesthesia and US guidance. 25% of the volume was replaced with 95% ethanol and then completely aspirated after 20–30 minutes.

No complications or deaths occurred. In all patients symptoms disappeared after treatment. In 15 out of 21 cases there was no evidence of residual cyst on US, computed tomography (CT) or magnetic resonance (MRI). In 6 patients with shorter follow-up, cysts showed a mean reduction in diameter of 50%. The mean follow-up was 18 months (range 6–60 months).

We conclude that PAS is easy with low risk for the patients and with good long-term results; it should therefore become the procedure of choice for simple hepatic cysts.

KEY WORDS: Interventional ultrasonography (US)-Liver cysts therapy-alcohol

### INTRODUCTION

The increased use of ultrasonography (US) has led to increased detection of simple hepatic cysts allowing better definition of their prevalence, which is now reported to be between 1% and 5%[1,2].

Although the majority of simple hepatic cysts are small and asymptomatic some of them are large and may cause symtoms; in these cases treatment is necessary. Until recently surgery was the only form of therapy, but the development of interventional ultrasound has led to alternative non-surgical techniques[3–6].

Previous personal experience with percutaneous alcohol sclerotherapy (PAS) of simple hepatic cysts had yielded good results[7]. Therefore we conducted a survey in some Italian centers with experience in interventional ultrasound to assess the efficacy of this therapeutic approach in a larger number of cases.

### MATERIAL AND METHODS

Data on 21 patients submitted to PAS from 1987 to 1991 were retrospectively reviewed. There were 6 men and 15 women with a mean age of 53 years (range 21–78). Cysts were single in 13 cases and multiple in 8. Cysts were located in the right hepatic lobe in 14 patients, in the left lobe in 3 and were bilobar in 4. The mean diameter was 9 cm (7–15 cm). 20 patients were treated because of abdominal pain and/or hepa-

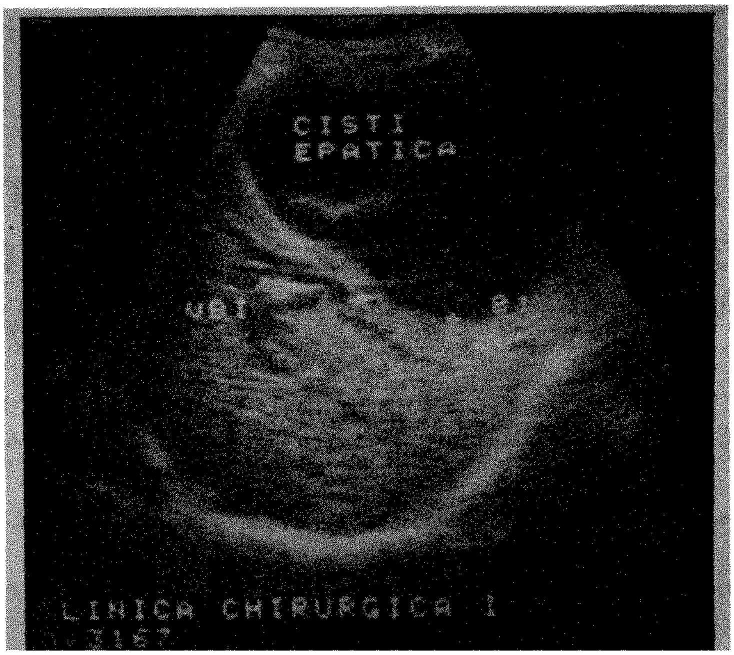

**Figure 1** Ultrasonographic appearance of a large simple hepatic cyst with a dilated intrahepatic biliary tree (VBI) due to cystic compression at the hepatic hilum.

tomegaly; the remaining patient presented with jaundice due to compression of intrahepatic bile ducts by a centrally located cyst (Figure 1). Pre-treatment diagnosis included liver US in all cases, computed tomography (CT) in 19 patients and magnetic resonance imaging of the liver (MRI) in the last 2 cases; in all patients complete serological testing of hidatidosis was performed. Liver and renal function tests were normal in all cases except for the patient with jaundice who had increased serum bilirubin and liver enzymes. All patients had a prothrombin time more than 60% and platelet count more than 80000/ml.

The procedure was performed at an outpatient following Bean and Rodan's technique[3]. After local anesthesia, under real-time ultrasound guidance with a 3.5 MHz convex probe, a 5–13 French pig-tail catheter was placed into the cyst using the Seldinger technique. In all cases samples of cyst fluid were sent for electrolyte determination and cytological, bacteriological and parasitological analysis. The cyst was completely aspirated and contrast medium injected into the residual cavity to rule out communication with the biliary tree or extravasation into the peritoneal cavity. After reaspiration of the contrast medium, 25% of the cyst volume was replaced with 95% ethanol which was left in the cyst for 20–30 minutes. During this time, the patient was rolled from side to side to allow better contact of the alcohol with the surfaces of the cyst cavity. The alcohol was then completely aspirated and the patient monitored by ultrasound. One instillation was judged to be sufficient. If the patient experienced severe pain during instillation of alcohol, the procedure was aborted and completed later.

### RESULTS

A mean of 450 ml of fluid was drained per puncture (range 140–630 ml). Only one puncture was performed for each patient; in the 8 patients with multiple cysts only the major cysts were treated with PAS. Liver function tests were never affected by therapy. Serum ethanol levels after PAS were not determined, however symptoms of alcohol intoxication were never detected. All bacteriological and parasitological tests were negative. In all cases neither malignant cells nor keratinized or squamous elements were found.

No major complications or deaths occurred; in one case abdominal pain was reported after injection of alcohol; the procedure was stopped for some minutes and completed after the pain had subsided. One patient had fever (38 °C) immediately after treatment which disappeared some hours later. Most patients were discharged on the day of treatment.

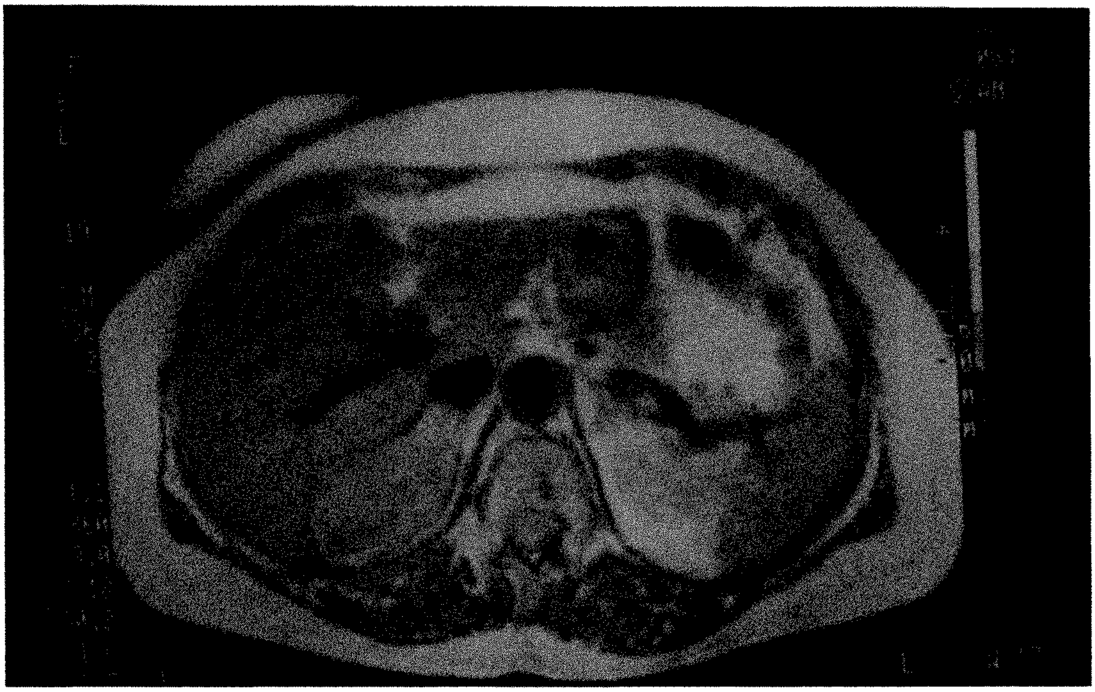

**Figure 2** Same cases as Figure 1. MRI shows no residual hepatic cyst 12 months after PAS.

The mean follow-up period was 18 months (range 6–60 months). In all patients the symptoms disappeared after treatment. In 15 out of 21 cases there was no evidence of a residual cyst on US, CT or MRI. In this group of patients mean follow-up was 22 months and mean duration of cyst disappearance was 17.6 months (range: 12–24 months). In the patient with jaundice before PAS, serum bilirubin levels and intrahepatic bile ducts at ultrasonography returned to normal immediately after treatment; at MRI there was no evidence of residual cyst 12 months after PAS (Figure 2). In 6 patients with shorter follow-up (6–9 months), cysts were still present; however all of them showed a mean reduction in diameter of 50% compared to pre-treatment values (Figure 3a, b).

### DISCUSSION

Simple cysts of the liver may present in different forms; they may be small or large, unilocular or multilocular and single (50% of cases) or multiple[1,2,8–10]. The more frequent signs and symtoms are hepatomegaly (54%), pain (33%), jaundice (9%) and sometimes nausea and vomiting[3]; these are usually due to cyst enlargement (reported in 10–15% of cases)[2,11], but more frequently (80–90%) they are asymptomatic.

Less than 5% of simple hepatic cysts develop acute complications such as torsion, intracavitary hemorrhage, infection or rupture[2,9,12,13].

Until a few years ago surgery was the only available treatment for symptomatic cysts[14] and included cyst marsupialization, drainage, partial or complete pericystectomy or hepatic resection[2,8,14]. Drainage may be external or internal with cysto-entero-anastomoses. Major problems have been reported with this approach because of cyst infection from retrograde contamination by enteric bacteria[8,14]. Some authors consider hepatic resection the treatment of choice for symptomatic simple hepatic cysts. In their opinion it is justified when enucleation is not possible and it also precludes relapse or degeneration[2,15,16]. Nevertheless most authors prefer the deroofing technique[8,17]. Recently some cases of laparoscopic deroofing of hepatic cyst were reported with good results; this new approach seems to reduce morbidity and hospital stay[18,19].

The increased use of interventional ultrasound has stimulated some alternative approaches to surgery. Simple percutaneous drainage was not effective because of the constant recurrence of cysts[4,5]. A number of sclerosing agents such as glucose, phenol, formaldehyde, and pantopaque have been used in percutaneous treatment of renal cysts and in a small number of cases

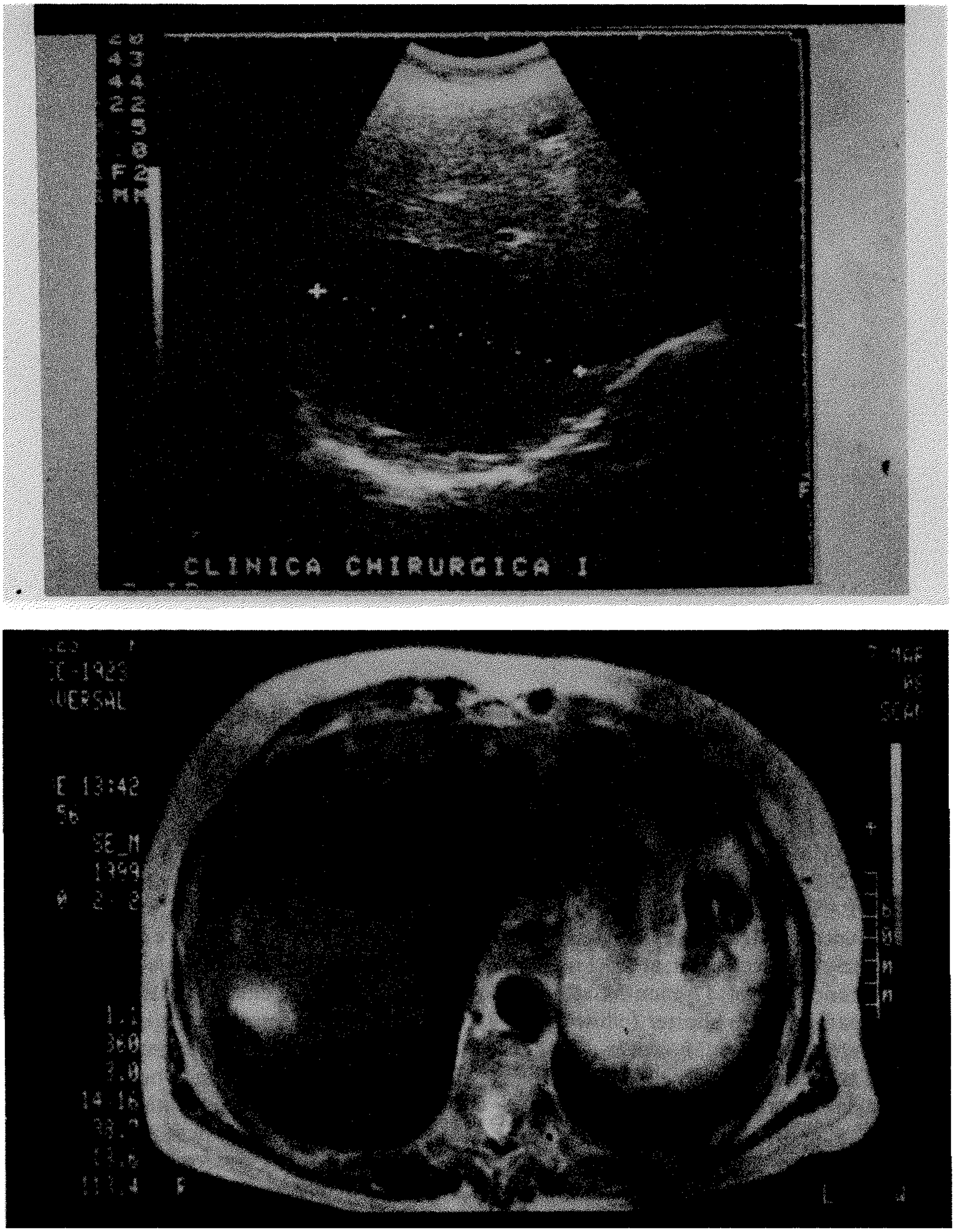

**Figure 3** a) Ultrasound of a large simple hepatic cyst before PAS. b) Cyst appearance 8 months after PAS.

of hepatic congenital cysts[3,6,20]. Results were often unsatisfactory with high recurrence rate and/or high toxicity[3,6,20]. A breakthrough came with the advent of alcohol sclerotherapy.

Alcohol sclerotherapy has already been used successfully in the treatment of benign renal cysts[21]. The aim is to induce a chemical cystitis with a secondary reactive sclerosis. Bean, Rodan and Trinkl *et al.* proposed percutaneous ethanol injection as a treatment for congenital hepatic cysts[3,22]. Their pre-liminary experience with 7 cases confirmed the effectiveness of this approach; the cysts completely disappeared after 6–18 months in all patients. Further similar experiences have been reported by Andersson *et al.*[20], Kairaluoma *et al.*[23] and Guglielmi *et al.*[24] for a total of 28 treated patients. These authors reported the disappearance of symptoms and near total disappearance of cysts with no mortality or major morbidity. Some patients experienced slight abdominal pain following PAS; only in one patient was the pain so severe as to stop the procedure. An elderly woman had symptoms of moderate alcohol intoxication after PAS.

In our experience ethanol injection under ultrasound guidance has proved to be a low-cost, safe and effective procedure. Results from our multicentre study confirm similar encouraging results from other authors. Mortality was nil and morbidity was negligible.

One of the most important problems with a percutaneous approach is the differential diagnosis of cystic hepatic lesions. Echinococcal cysts may mimic simple cysts in the case of early, type I, cysts[11,25,26]. In most cases imaging techniques, such as US and CT identified echinococcal cysts. It is mandatory to accurately assess the clinical history and to per- form appropriate and sensitive serological tests[27,28].

Hepatic cystadenoma and cystadenocarcinoma are rare cystic tumors[11,29,30]. Generally these neoplasms have a typical appearance on ultrasound and CT with a thick and sometimes calcified wall, septations and parietal growths which are hyperechoic on US or hyperdense with contrast medium on CT[1,2,29,31,32]. These morphological aspects associated with a progressive increase in volume permits an accurate diagnosis even if differentiation between cystadenoma and cystadenocarcinoma is difficult[29].

Cystic hepatic metastases account for 3% of all metastatic lesions of the liver; US, CT and an accurate clinical history are usually sufficient to differentiate these lesions from simple hepatic cysts[2,32].

A cytological examination of the fluid content should be obtained especially when the definite diagnosis cannot be made[32,33]. Fine Needle Aspiration (FNA) appears to be a safe and accurate procedure. Physicochemical and parasitological analysis of fluid content is useful to diagnose echinococcal diseases and percutaneous puncture of these lesions seem to be safe[27,28,34,35]. FNA may be important in detection of rare complications of a simple hepatic cyst such as neoplastic degeneration of the epithelium to squamous cell carcinoma and adenocarcinoma; in these infrequent cases the findings of squamous, keratinized cells in aspirated fluid associated with invasive growth appearance on US and CT is suggestive of malignancy[36–39]. CEA measurement on cystic fluid may be able to differentiate between cystic hepatic neoplasms on simple hepatic cysts[40].

In our experience the high diagnostic accuracy of imaging techniques and FNA allows a percutaneous approach in the treatment of simple hepatic cysts; moreover previous lack of complications in treatment of hepatocellular carcinoma and hydatid liver cyst with percutaneous ethanol injection[35,41] suggests that an accidental instillation of alcohol into a malignant or parasitic cystic liver lesion would not harm the patient. Injection of contrast medium under radiological contrast into the cystic cavity proved to be sufficient to rule out cyst communications with the biliary tree (an absolute contraindication to PAS)[3,6,20,23].

In conclusion our multicentric study proved that PAS can be performed with no mortality and negligible morbidity in centers with experience in interventional ultrasound. Therefore this technique should be proposed as the first choice treatment for symptomatic simple hepatic cysts.

### REFERENCES

1. Benhamou, J. P. (1989) Les kystes non parasitaires du foie. La Revue du Praticien, **18**, 1602–3.
2. Sanchez, H., Gagner, M., Rossi, R. L., Jenkins, R. L., Lewis, W. D., Munson, J. L., Braasch, J. W. (1991) Surgical management of nonparasitic cystic liver disease. *Am. J. Surg.*, **161**, 113–9.
3. Bean, W., Rodan, B. A. (1985) Hepatic cysts: treatment with alcohol, *AJR*, **144**, 237–41.
4. Roemer, C. E., Ferrucci, Jr. J. T., Mueller, P. R., Simeone, J. F., Van Sonnenberg, E., Wittenberg, J. (1981) Hepatic cysts: diagnosis and therapy by sonographic needle aspiration. *AJR*, **136**, 1065–70.
5. Saini, S., Mueller, P. R., Ferrucci, Jr. J. T., Simeone, J. F., Wittenberg, J., Butch, R. J. (1983) Percutaneous aspiration of hepatic cysts does not provide definitive therapy. *AJR*, **141**, 559–60.
6. Goldstein, H. M., Carlyle, D. R., Nelson, R. S. (1976) Treatment of symptomatic hepatic cyst by percutaneous instillation of pantopaque. *AJR*, **127**, 850–3.
7. Montorsi, M., Torzilli, G., Spiropoulos, J., Zago, M., Poggio, A. (1990) Alcolizzazione percutanea delle cisti epatiche semplici nonparassitarie. Proceedings "VI Congresso Nazionale della SIATEC", Bologna (Italy), 853–7.
8. Huguier, M., Paquet, J. C., Roland, J., Houry, S., Lacaine, F. (1990) Biliary cysts of the liver. *Dig. Surg.*, **7**, 93–6.
9. Davis, C. K., Schoffatall, R. O., Glass, T. F. (1981) Fatal complication of hepatic cystic disease. *South. Med. J.*, **74**, 1409–11.
10. Clark, D. D., Marks, C., Bernhard, V. M., Bunkfeldt, F. (1967) Solitary hepatic cysts. *Surgery*, **51**, 687–93.
11. Bruneton, J. N., Eresue, J., Caramella, E., Drouillard, J., Roux, P., Fenart, D. (1983) Les kystes congenitaux du foie en ecographie. *J. Radiol.*, **64**(8–9), 471–6.

12. Akriviadis, E. A., Steindel, H., Ralls, P., Redeker, A. G. (1989) Spontaneous rupture of nonparasitic cyst of the liver. *Gastroenterology*, **97**, 213–5.
13. Ayyash, K., Hadded, J. (1988) Spontaneous rupture of a solitary nonparasitic cyst of the liver: Case report. *Acta Chir. Scand.*, **154**, 213–4.
14. Fernandez, M., Cacioppo, J. C., Davis, R. P., Nora, P. F. (1984) Management of solitary nonparasitic liver cysts. *Am. Surg.*, **50**(4), 205–8.
15. Morelli, N., Pellicci, R., Taviani, M., Lo. Casto, M. R., Verna, A., Tommasi, G. V., Maritato, F., Cariati, E. (1991) Il trattamento delle cisti solitarie del fegato. *Chirurgia*, **4**, 242–7.
16. Rumi, A., Arcidiaco, M., Testone, G., Soffiantini, G. (1991) Cisti disontogenetiche del fegato. Quando e perchèla resezione epatica. *Chirurgia*, **4**, 596–600.
17. Litwin, D. E. M., Taylor, B. M., Langer, B., Greig, P. (1987) Nonparasitic cysts of the liver: the case for conservative surgical management. *Ann. Surg.*, **205**(1), 45–8.
18. Paterson-Brown, S., Garden, O. J. (1991) Laser-assisted laparoscopic excision of liver cyst. *Br. J. Surg.*, **78**, 1047.
19. Katkhouda, N., Fabiani, P., Benizri, E., Mouiel, J. (1992) Laser resection of a liver hydatid cyst under video laparoscopy. *Br. J. Surg.*, **79**, 560–1.
20. Andersson, R., Jeppsson, B., Lunderquist, A., Bengmark, S. (1989) Alcohol sclerotherapy of non parasitic cysts of the liver. *Br. J. Surg.*, **76**, 254–5.
21. Bean, W. J. (1981) Renal cysts: treatment with alcohol. *Radiology*, **138**, 329–31.
22. Trinkl, W., Sassaris, M., Hunter, F. M. (1985) Nonsurgical treatment for symptomatic nonparasitic liver cyst. *Am. J. Gastroenterol.*, **80**(11), 907–11
23. Kairaluoma, M. I., Leinonen, A., Stahlberg, M., Paivansalo, M., Kiviniemi, H., Siniluoto, T. (1989) Percutaneous aspiration and alcohol sclerotherapy for symptomatic hepatic cysts. *Ann. Surg.*, **210**(2), 208–15.
24. Guglielmi, A., Veraldi, G. F., Furlan, F., Soardi, G. A., de Manzoni, G. (1991) La terapia percutanea ecoguidata delle cisti epatiche disontogenetiche. *Ann. Ital. Chir.*, **LXII**(1), 13–7.
25. Gharbi, H. A., Hassine, W., Brauner, M. W., Dupuch, K. (1981) Ultrasound examination of the hydatid liver. *Radiology*, **139**, 459–63.
26. Lewall, D. B., McCorkell, S. J. (1985) Hepatic echinococcal cysts: sonographic appearance and classification. *Radiology*, **155**, 773–5.
27. Livraghi, T., Bosoni, A., Giordano, F., Lai, N., Vettori, C. (1985) Diagnosis of hydatid cyst by percutaneous aspiration: value of electrolyte determinations. *J. Clin. Ultrasound*, **13**, 333–7.
28. Diakoumakis, E. E., Weinberg, B., Vieux, U., Seife, B. (1985) Varied sonographic patterns in echinococcus liver disease. *J. Clin. Ultrasound*, **13**, 627–31.
29. Korobkin, M., Stephens, D. H., Lee, K. K. T., Stanley, R. J., Fishman, E. K., Francis, I. R., Alpern, M. B., Rynties, M. (1989) Biliary cystadenoma and cystadenocarcinoma: CT and sonographic finding. *AJR*, **153**, 507–11.
30. Lewis, W. D., Jenkins, R. L., Rossi, R. L., Munson, L., ReMine, S. G., Cady, B., Braasch, J. W., Mc Dermott, W. V. (1988) Surgical treatment of biliary cystoadenoma. A report of 15 cases. *Arch. Surg.*, **123**, 563–68.
31. Akwari, O. E., Tucker, A., Seigler, H. F., Itani, K. M. F. (1990) Hepatobiliary cystadenoma with mesenchymal stroma. *Ann. Surg.*, **211**(1), 18–27.
32. Federle, M. P., Filly, R. A., Moss, A. A. (1981) Cystic hepatic neoplasm: complementary roles of CT and sonography. *AJR*, **136**, 345–8.
33. Barnes, P. A., Thomas, J. L., Bernardino, M. E. (1981) Pitfalls in the diagnosis of hepatic cysts by computed tomography. Radiology, **141**, 129–133.
34. Ben Amor, N., Gargouri, M., Gharbi, H. A., Golvan, Y. J., Ayachi, K., Kchouk, H. (1986) Essai de traitement par ponction des kystes hydatiques abdominaux inoperables. *Ann. Parasitol Hum. Comp.*, **61**, 689–92.
35. Filice, C., Pirola, F., Brunetti, E., Dughetti, S., Strosselli, M., Foglieni, C. S. (1990) A new therapeutic approach for hydatid liver cysts. Aspiration and alcohol injection under sonographic guidance. *Gastroenterology*, **98**, 1366–8.
36. Greenwood, N., Orr WMcN. (1972) Primary squamous-cell carcinoma arising in a solitary nonparasitic cyst of the liver. *J. Pathol.*, **107**, 145–8.
37. Homer, L. W., White, H. J., Read, R. C. (1968) Neoplastic transformation of Von Meyenburg complexes of the liver. *J. Pathol. Bacteriol.*, **96**, 499–502.
38. Gresham, G. A., Rue, L. W. (1985) Squamous cell carcinoma of the liver. *Hum. Pathol.*, **16**, 413–6.
39. Lynch, M. J., McLeod, M. K., Weatherbee, L., Gilsdorf, J. R., Guice, K. S., Eckhauser, F. E. (1988) Squamous cell cancer of the liver arising from a solitary benign nonparasitic hepatic cyst. *Am. J. Gastroenterol.*, **83**(4), 426–31.
40. Pinto, M. M., Kaye, A. D. (1989) Fine needle aspiration of cystic liver lesions. Cytologic examination and carcinoembryonic antigen assay of cyst contents. *Acta. Cytol.*, **33**(6), 852–6.
41. Livraghi, T., Bolondi, L., Lazzaroni, S., Marin, G., Morabito, A., Rapaccini, G. L., Salmi, A., Torzilli, G. (1992) Percutaneous ethanol injection in the treatment of hepatocellular carcinoma in cirrhosis. A study on 207 patients. *Cancer*, **69**(4), 925–9.

